# Chemical Mechanisms of Prebiotic Chirality Amplification

**DOI:** 10.34133/2020/5689246

**Published:** 2020-08-04

**Authors:** Konstantin P. Bryliakov

**Affiliations:** ^1^Novosibirsk State University, Pirogova 1, Novosibirsk 630090, Russia; ^2^Boreskov Institute of Catalysis, Pr. Lavrentieva 5, Novosibirsk 630090, Russia

## Abstract

This *review article* surveys the recent experimental findings that suggest alternative chemical models of directed chirality amplification at the early, prebiotic Earth. It is believed that the chirality amplification step followed the initial emergence of small enantiomeric imbalance and preceded (as a necessary condition) the occurrence of homochiral biopolymers, assembled from enantiomerically pure building blocks. This work focuses on the chemical nature of possible mechanisms of primordial chirality enhancement, without going into detail of the preceding and subsequent phases of origination of biological homochirality and life on Earth. These mechanisms are discussed through the prism of integrity of biological natural selection and chemical kinetic selection.

## 1. Introduction

Chirality is an inherent property of living matter; moreover, the existence of chirality has been considered as a necessary condition for the emergence of life [1], which complex phenomenon—in many of its aspects—is likely to become the central object of natural sciences of the 21^st^ century. For evident reasons, we cannot observe experimentally the emergence of life from simple feedstock molecules, which, according to estimates, could be the case between 4.3 and 3.8 billion years ago [2–4]. At the current level of our knowledge, we cannot reliably reproduce it in the laboratory, either. We can only try to reconstruct or model some of its elements, yet so far without solid criteria to adhere to in such recapitulations.

The emergence of life has been inseparably linked with the emergence of biological homochirality (*L*-amino acids, *D*-sugars) [5–8]. To date, the *biotic* theories, hypothesizing that homochirality emerged as inevitable consequence of the evolution of living matter [9, 10], have been virtually abandoned (for being not amenable to testing in any scientific sense). In turn, *abiotic* theories have prevailed, assuming that life at its origin already had available chiral molecules possessing sufficient enantiomeric homogeneity. This homochirality could not have originated without emergence of some initial asymmetric bias (albeit small) in the primordial molecular environment, followed by amplification of enantiomeric excess up to the level sufficient for constructing biopolymers, capable of replicating their homochirality.

Abiotic theories can be subdivided into *stochastic* and *deterministic* ones, assuming that biological chirality started “by chance” or “by law,” respectively [9, 10], with the latter apparently dominating at the moment. Furthermore, the standpoint that the initial chirality protosource was introduced from without has received the major experimental and theoretical attention, given that spatial objects contain nonracemic organic matter in abundance (noticeably, the majority of nonracemic amino acids found in meteorites have *L*-configuration in preference [11–15]). Within this paradigm, the stage of *prebiotic* chirality amplification (either up to homochiral state or not) is thought of as essential step of terrestrial evolution [6, 10].

In the last decades, different hypotheses on the possible mechanisms of prebiotic, or primordial, chirality amplification were put forward and criticized from different perspectives [2, 5, 16–22]. This contribution is aimed at considering the models relying on far-from-equilibrium *chemical mechanisms* of enantiomeric enrichment, essentially leaving to separate discussions crystallization-induced chirality amplifications [23], typically considered in connection with the “chance” paradigm [9]. For the purposes of this work, we focus on catalysis-based mechanisms, in agreement with the prerogative of a (chiral) catalyst molecule to mediate a number of single asymmetric transformations, affording plenty of chiral product molecules.

Recently, Davankov pointed out the following essential characteristics of the chemical systems that claim to model or recreate primordial terrestrial chirality amplification: they should “function *efficiently* and *unidirectionally* in a complex concentrated *aqueous* broth” [2]. To date, there apparently have been no chemical systems that would fit ideally within these requirements. On the other hand, if the origins of biological chirality were governed “by law,” in principle, it could not a priori be excluded that chirality enrichment processes could take place elsewhere in the Universe before the resulting nonracemic organic matter approached our planet. So we might expect that model systems should conform to some universal regularities, irrespective of the conditions that hypothetically existed at the primitive Earth.

This contribution discusses experimental findings suggesting possible model catalytic mechanisms of prebiotic chirality amplification. Some of those set off the significance of *dynamic nonlinear effects* (DNLE) in asymmetric catalysis [24] for the overall discussion of the origins of biological chirality. A few other recent intriguing hypotheses, going without the involvement of dynamic nonlinear effects, are considered as well.

## 2. Asymmetric Autocatalytic Reaction

**Figure 1:**
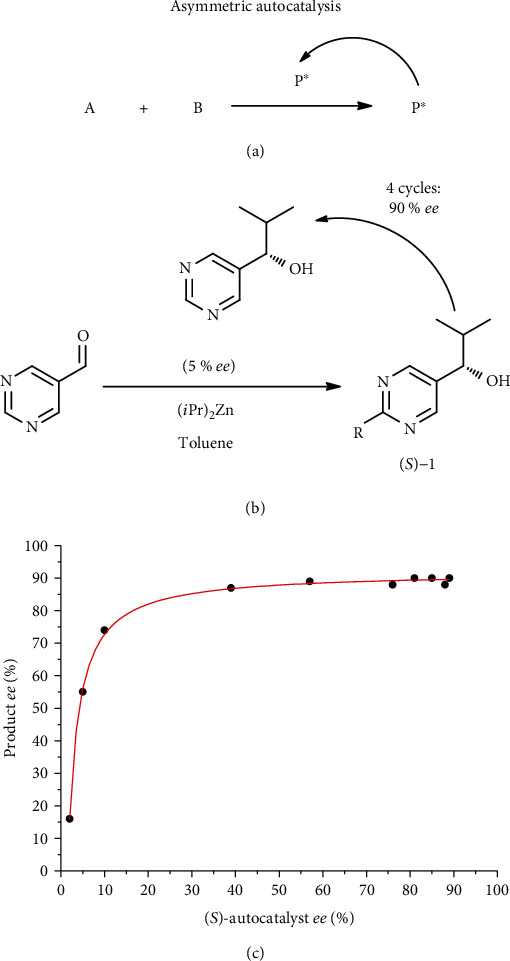
Asymmetric autocatalytic processes, where A is the substrate, B is the reactant, C is the catalyst, and P∗ is the chiral product (a). Autocatalytic asymmetric diethylzinc addition to pyrimidine-5-carbaldehyde (the Soai reaction) with amplification of enantiomeric excess (b). Relationship between the enantiomeric purity of the initial catalyst and of the newly formed product in the Soai reaction, according to Ref. [19] (c).

Asymmetric autocatalytic reaction is a chemical process in which a chiral molecule mediates its own formation from achiral (prochiral) reagents in a cyclic process, thus playing the role of chiral autocatalyst (Figure 1(a)). Formally, *asymmetric autocatalysis* is a DNLE, corresponding to a specific case of catalytic *asymmetric autoinduction*, a more general phenomenon consisting in the effect of the chiral reaction product on the stereochemical course of the asymmetric reaction [24–27].

The most spectacular class of asymmetric autocatalytic reactions was discovered by Soai with coworkers in 1990 [28]; crucially, in 1995, the same group reported the first example of autocatalytic reaction *with amplification of enantiomeric excess* (Figure 1(b)) [29]. When examining the addition reaction of diisopropylzinc to pyrimidine-5-carboxaldehyde (Figure 1(b)), the authors loaded the autocatalyst, (*S*)-**1**, having initial 5% *ee*, which resulted in the formation of the product having higher enantiomeric purity of 39% *ee*. The latter was introduced as catalyst for the next, larger-scale run, also ending up with chirality amplification, etc. After the 4^th^ run, the newly formed product (*S*)-**1**, having 90% *ee*, was obtained [29].

**Figure 2:**
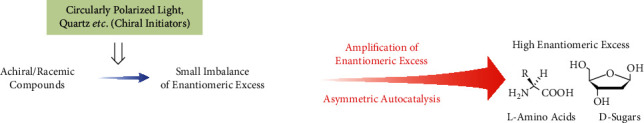
Proposed asymmetric autocatalysis-assisted route to homochirality. Reprinted from Ref. [20] (https://pubs.acs.org/doi/abs/10.1021/ar5003208). Copyright 2014 American Chemical Society; further permissions related to the material excerpted should be directed to the ACS.

In the context of the present work, the Soai reaction provides an attractive chemical model for the reproducible amplification of chirality, starting from initial enantiomeric imbalance, which could be as low as 0.00005% *ee* [30] or perhaps even lower [31]. It is thus compatible with the hypothesis of extraterrestrial introduction of organic matter with initial small enantiomeric imbalance (Figure 2). It is important to notice that the asymmetric bias could be introduced to the Soai reaction not only by nonracemic chiral product/autocatalyst but also by extraneous chiral additives, such as quartz, chiral inorganic crystals, helical silica gel, small chiral (including isotopically chiral) organic molecules [20, 32–34], and the abiogenous chiral amino acids obtained from the Murchison meteorite [35], simulating the “chiral triggers” of extraterrestrial origin.

Moreover, the asymmetric Soai reaction also matches the stochastic hypothesis of the abiotic origination of chirality. Indeed, starting from completely achiral conditions, spontaneous symmetry breaking in the Soai reaction has been documented, demonstrating quasistochastic distribution of the (*R*)- and (*S*)-enantiomers of the product [36–38]. Noticeably, spontaneous mirror symmetry breaking, with subsequent amplification of enantiomeric excess, is not specific for the Soai reaction: it has also been reported for the asymmetric autocatalytic Mannich and aldol reactions [39–42], providing further indirect support of the viability of asymmetric autocatalytic reactions as possible protagonists of the discussion on the mechanisms of primordial chirality amplification.

The criticism of the asymmetric autocatalytic model focuses on the high sensitivity of the outcome of the Soai reaction (which so far has been the only autocatalytic reaction ensuring *significant* enhancement of enantiomeric excess) to external additives (even not necessarily chiral [43]), which again puts “chance” in the forefront, bringing into question the capability of the Soai system to function unidirectionally under nonideal reaction conditions. Another point is that organometallic Soai reaction, yet extremely efficient, requires strictly nonaqueous environment, which is not compatible with the presumably partially aqueous environment of the early Earth by the end of the abiotic period of its history.

## 3. Reaction with Asymmetric Autoamplification

**Figure 3:**
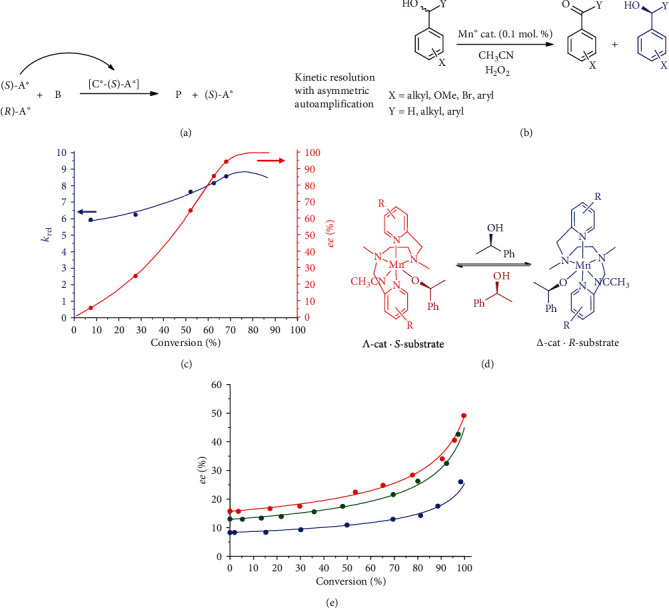
Kinetic resolution with asymmetric autoamplification, where A is the substrate, B is the reactant, C is the catalyst, P is the product, and asterisks indicate chiral molecules (a). Oxidative kinetic resolution of secondary alcohols in the presence of chiral Mn catalyst (b). Plots of *k*_rel_ (blue; experiment: circles, theory: line) and *ee* (red; experiment: circles, theory: line) vs. substrate (1-*p*-tolylethanol) conversion (c). Dynamic control of conformationally flexible catalyst's chirality upon substrate coordination (d). Substrate enantiomeric enrichment in the oxidative kinetic resolution of scalemic (*S*)-1-phenylethanol in the presence of conformationally flexible Mn catalyst, starting from substrates with differing *ee*.

An alternative, nonautocatalytic (but also DNLE-based) hypothesis of prebiotic chirality amplification was suggested by the discovery of the novel dynamic nonlinear effect, termed asymmetric autoamplification (Figure 3(a)), in the oxidative kinetic resolution of secondary alcohols (Figure 3(b)), catalyzed by chiral *bis*-amino-*bis*-pyridine manganese complexes [44, 45]. The crucial feature of asymmetric autoamplification is that the stereochemical course of the reaction is affected by the chiral *substrate* itself, via interaction with the chiral catalyst. The formal reaction product, ketone, is not chiral. In effect, as the reaction proceeds, the enantiomeric purity of the substrate increases, thus increasing the selectivity factor of kinetic resolution (Figure 3(c)). As shown from the above, kinetic resolution reactions are the “native habitat” of asymmetric autoamplification phenomena.

However, asymmetric autoamplification itself is not sufficient basis for designing a model chemical mechanism of chiral amplification. For this purpose, it had to meet another interesting effect, dynamic control of the catalyst chirality by the chiral substrate [46, 47]. This was achieved by using manganese catalysts bearing conformationally flexible achiral *bis*-amino-*bis*-pyridine ligands. Those octahedral chiral-at-metal manganese complexes could adopt two different stereoconfigurations, *Δ* and *Λ*. In the absence of any chiral molecules, the equilibrium mixture of the *Δ* and *Λ* isomers is racemic. However, chiral secondary alcohol can control the stereoconfiguration of the Mn complex via coordination, thus shifting this equilibrium towards one of the stereoisomers (Figure 3(d)). Taking into account that the catalyst enantiomers have opposite stereoselectivities in kinetic resolution, substrate coordination thus determines the net stereoselectivity of the catalyst system [47].

**Figure 4:**
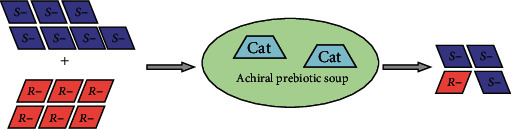
Autoamplification/dynamic chirality control model of prebiotic chirality enhancement. “*R*-” and “*S*-” are enantiomeric substrates, “Cat” is catalyst. Reprinted from Ref. [47].

In effect, over the course of oxidation of an initially nonracemic (scalemic) mixture of the (*R*)- and (*S*)-alcohols, monotonic enantiomeric enrichment is the case (Figure 3(e)). The initially prevailing substrate enantiomer itself controls the stereochemical outcome of the reaction, without action of any exogenous chirality sources, and eventually “outcompetes” the other enantiomer in the concurrent catalyzed oxidation reaction. This may be regarded as a prototype of through-competition chemical evolution model, having direct analogy with the natural selection-driven biological evolution [48]. The model reaction operates predictably and unidirectionally in aqueous-organic medium, in line with the conditions expected to exist in the primitive environment. The corresponding vision of abiotic chirality self-enrichment is presented in Figure 4, assuming (extraterrestrial?) introduction of a scalemic substrate mixture with small enantiomeric imbalance, which, upon contact with achiral prebiotic environment, triggers its own kinetic resolution with self-controlled enantioenrichment.

## 4. Asymmetric Catalytic Reaction with “Hyperpositive” Nonlinear Effect

**Figure 5:**
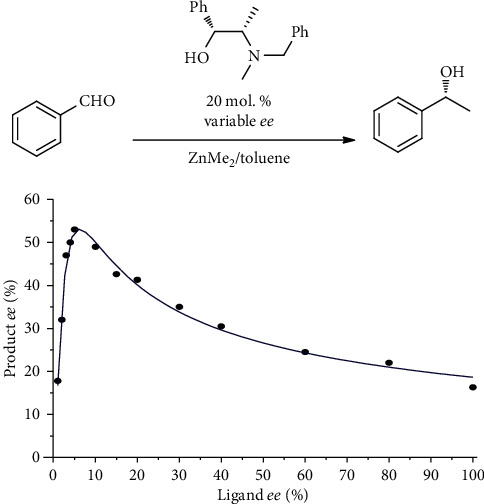
Nonconventional nonlinear effect (NLE) in the (-)-NBE-catalyzed addition of ZnMe_2_ to benzaldehyde.

Very recently, Geiger with coworkers reported an asymmetric catalytic reaction with unusual behavior [49], which suggested an idea of alternative, apparently not DNLE-based, chirality amplification mechanism. The authors discovered that the enantioselectivity of dialkylzinc addition to benzaldehyde, in the presence of nonenantiopure catalyst (1*R*,2*S*)-*N*-benzylephedrine ((-)-NBE), counterintuitively *increased* with *decreasing* catalyst enantiomeric purity (Figure 5). In fact, at 5% *ee* of the (-)-NBE chiral auxiliary, the enantioselectivity reached a maximum, exceeding the enantioselectivity of the enantiopure catalyst. Such behavior is reminiscent of the “hyperpositive” *nonlinear effect* (NLE), theoretically predicted by Guillaneux et al. [50], yet presumably has different origin [49, 51].

The authors explained the observations by the existence of two enantiodivergent pathways, in which both monomeric (more enantioselective) and homochiral dimeric (less enantioselective) (-)-NBE-ZnMe complexes catalyzed the reaction, with different enantioselectivities, while the heterochiral dimeric species did not intervene the catalyzed reaction, precipitating down the solution [49]. This simplified mechanistic landscape does not take into account [51] possible intervention of asymmetric autoinduction (which is archetypical of dialkylzinc to aldehydes addition) [25, 26] into the observed enantioselectivity, as well as that of isoinversion behavior [52], leading to the unusual temperature dependence of enantioselectivity [49]. Although the detailed mechanism of the reaction has remained not entirely clear, the experimental observations of the authors provide the first, spectacular example of a reasonably highly enantioselective process, occurring with the assistance of a catalyst which has rather low yet statistically meaningful enantiomeric excess (i.e., similar to those documented for extraterrestrial organic substances obtained from meteorites). The organometallic nature of the present reaction, and thus its incompatibility with aqueous environment, does not belittle the high fundamental significance of these findings for the discussion of the emergence of biological homochirality.

## 5. Asymmetric Catalytic Reaction without Nonlinear Effects

**Figure 6:**
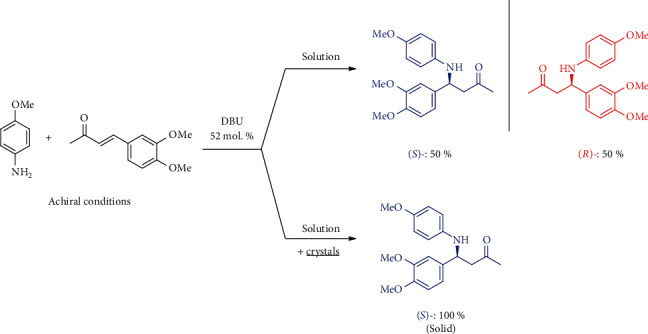
Aza-Michael reaction under homogeneous solution conditions and in the presence of enantiopure crystals.

Vlieg with coworkers suggested an alternative mechanism, combining a catalytic reaction and crystallization-assisted deracemization. In particular, they showed that 1,8-diazabicyclo[5.4.0]undec-7-ene (DBU) catalyzed both the forward aza-Michael reaction and the backward one (Figure 6) [53]. Under homogeneous solution conditions, this reaction leads to a racemic mixture of products. However, in case there are enantiopure nuclei of crystals of the product in the system, they facilitate crystallization of the same enantiomer (let it be (*S*)-enantiomer as shown in Figure 6), thus leading to depletion of the solution phase in this product enantiomer. This enantiomeric imbalance does not persist since the DBU catalyzed backward reaction, followed by the forward one, effectively leads to deracemization of the excess (*R*)-enantiomer in solution.

Let us now consider that there is a mixture of separate enantiopure single crystals (that is, conglomerate crystals), with the (*S*)-crystals being present in excess. Given the equilibrium between the solid phase and solution, the crystals of the (*R*)-enantiomer can redissolve and undergo deracemization via backward reaction, and crystallize again in the (*S*)-form, thus eventually leading to the formation of crystalline (*S*)-enantiomer having 100% *ee*. In this case, the overall process is rate limited by dissolution of crystals, which can be accelerated by lowering the reactant concentrations [53].

Although Vlieg with coworkers did not add any crystals on purpose, working under deliberately achiral conditions and relying on spontaneous crystallization of the product, preferential formation of enantiopure (*S*)-product crystals (in 39 experiments) over the (*R*)-product (in 29 experiments) was documented. The mechanism of this bias cannot be explained by statistical fluctuations of *ee* or difference between size distributions of enantiomers crystals; the authors invoked possible presence of some unidentified chiral impurities, which could inhibit nucleation of the product crystals or the catalyzed processes in solution [53].

These results demonstrate the possibility of attaining enantiopure product from achiral reactants in a *racemoselective* reversible catalytic reaction, coupled with precipitation-induced chiral amplification, possibly biased by a minor chiral impurity, without action of any nonlinear effects. More recently, analogous approach has been reproduced in the absolute asymmetric Strecker synthesis of *α*-aminonitriles in a water-methanol solution [54]. In the latter case, quasistochastic formation of (*R*)- and (*S*)-products has been observed (37 vs. 36 times), apparently reflecting the absence of intervention of any external chiral effects.

## 6. Conclusions

Summarizing the discussion above, we would like to point out that only the models of chirality amplification based on DNLEs, i.e., on asymmetric autocatalysis and asymmetric autoamplification/dynamic chirality control [33, 34, 42, 47], stand close to the expected conditions for mimicking natural selection in chemical systems [55]. The DNLE-based models, dealing with far-from-equilibrium chemical systems, operate by analogy with competition-based biological processes (which are fundamentally irreversible) [48, 55, 56], while the models exploiting crystallization-assisted chiral amplification [53, 54] or “hyperpositive” nonlinear effects [49] lack this similarity. From the mathematical perspective, the former chemical systems obey the rules similar to those of natural selection [48, 56], which allows one entity (enantiomer), competing for the same chemical stuff, to drive the other competitor out completely and dominate the available space. From the systems theory perspective, this is realized via positive feedback coupling, further assisting the process as it proceeds—which, for a chemical process, is possible in case its rate equations are fundamentally nonlinear. The other mechanisms considered herewith lack this feedback coupling; besides, the reversible step, associated with deracemization, has no direct analogy (“resurrection”) in biological life.

At the current level of understanding, it cannot be firmly established that the process which afforded the enantiomerically enriched (bio)monomers was an abiotic prototype of biological natural selection. Still, there is no theoretical obstacle which prevents natural selection from having an equivalent (kinetic selection) in nonliving systems [55]; we could expect this, given a single driving force operating at both the molecular level and the biological level—the drive toward greater kinetic stability [57]. Also, it has remained unknown whether biomonomers achieved complete or partial enantiopurity before they polymerized into enantioenriched biopolymers, capable of self-replication [58]. These unsolved problems at the interface of chemical and biological perceptions of the complex phenomenon of life persist, thus continuing to be a challenge and inspiration for both disciplines, as well as for natural sciences in general. Hopefully, the present brief discussion of recent findings can add freshness to the area and contribute to the polemic on the chemical origins of life [55–59].
